# Supplementary material for: Tunable Self‐Emulsification Via Viscoelastic Control of Marangoni‐Driven Interfacial Instabilities
Source: Small Methods. 2025 Jul 16;10(1):2500749. doi: 10.1002/smtd.202500749 (PMC12790379; doi:10.1002/smtd.202500749)
Supplement: Supplementary file 1 — Supporting Information [file SMTD-10-2500749-s003.pdf]

Tunable self-emulsification via viscoelastic control of Marangoni-driven interfacial instabilities

Christoph Haessig[1,2], Mehdi Habibi[3], and Uddalok Sen*[1]

[1]Physical Chemistry and Soft Matter group, Wageningen University and Research, 6708 WE Wageningen, The Netherlands
[2]Department of Chemical Engineering, KU Leuven, 3001 Leuven, Belgium
[3]Laboratory of Physics and Physical Chemistry of Foods, Wageningen University and Research, 6708 WG Wageningen, The Netherlands

SUPPLEMENTARY INFORMATION

Density measurements

Densities were determined by weighing at least three 100 µL samples at 19 °C using a precision analytical laboratory balance (Mettler-Toledo GmbH) with an accuracy of 0.1 mg. The density of sunflower oil (ρ_o) was measured as 940 kg/m^3, while the density of the mother droplet (ρ_d) remained effectively constant across polymer concentrations. Accordingly, for all analyses, ρ_d was approximated as 930 kg/m^3 for PEO1M and 940 kg/m^3 for PEO4M.

Interfacial tension measurements

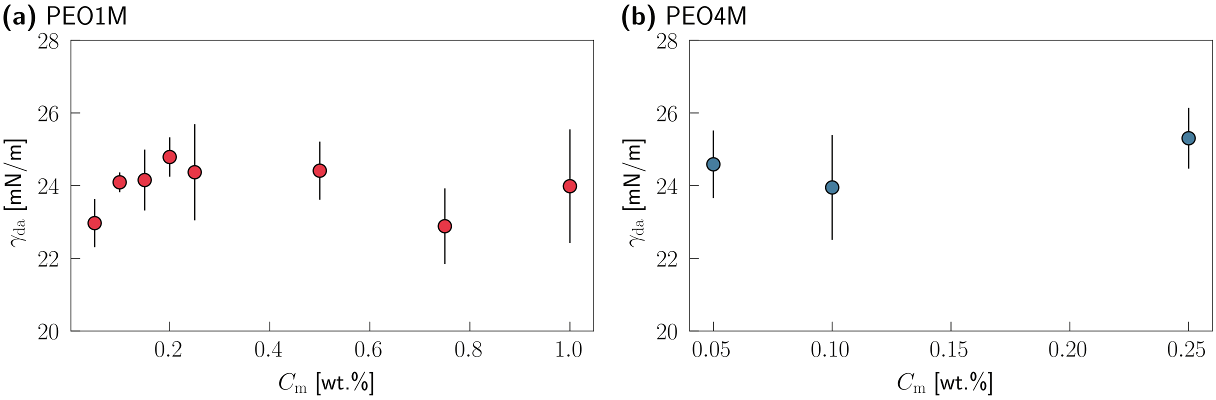

Figure S1: **Interfacial tension measurements.** Variation of drop-air interfacial tension coefficient, γ_{da}, with polymer concentration, C_m, for **(a)** PEO1M and **(b)** PEO4M. The discrete markers denote the mean of at least three independent experimental realizations while the error bars indicate ± one standard deviation.

Interfacial tension coefficients were obtained using the pendent drop method on a commercial drop shape analyzer (DSA 100E, Krüss GmbH). Images of axisymmetric droplets suspended from a hydrophobic Teflon-coated stainless steel needle (inner diameter = 0.25 mm, Nordson EFD) were recorded in both air and sunflower oil environments. The interfacial tension coefficients were extracted using the "Pendent_Drop" plugin[1] in the open-source image analysis software Fiji[2]. All measurements were performed at a temperature of 19 °C and in triplicate.

The interfacial tension coefficient of the sunflower oil-air interface (γ_{oa}) was determined to be 30.5 mN/m. Measurements (see figure S1) indicated that both the drop-oil (γ_{do}) and drop-air (γ_{da}) interfacial tension coefficients were effectively independent of the polymer concentration, C_m. Hence, we consider γ_{da} = 24 mN/m and γ_{do} = 4 mN/m for all calculations in the present study.

*uddalok.sen@wur.nl, ORCID: 0000-0001-6355-7605

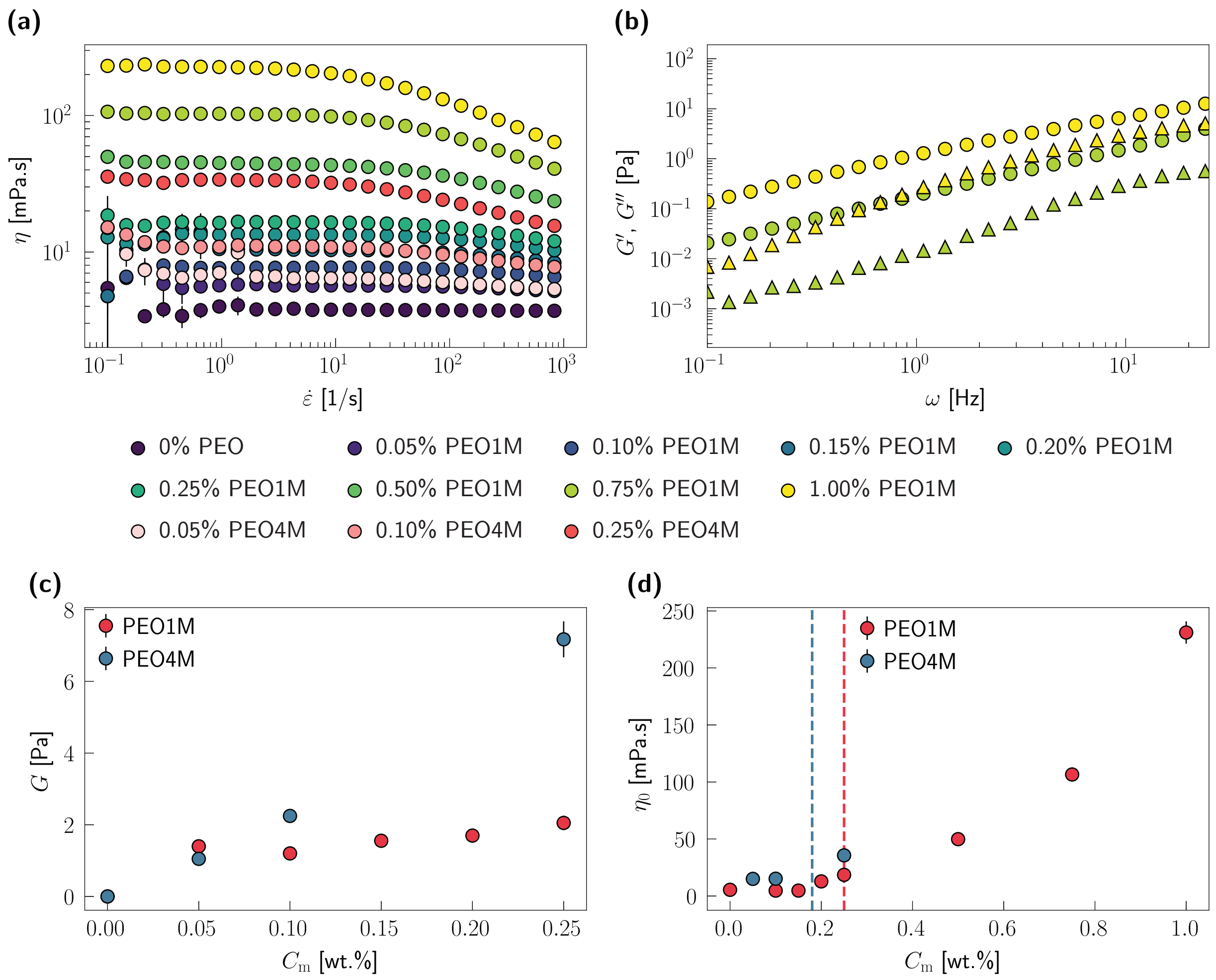

Figure S2: **Rheological characterization. (a)** Variation of shear viscosity, η, with shear rate, $\dot{\varepsilon}$, for different polymer concentrations. **(b)** Representative frequency (ω) sweep measurements, at 10% amplitude and for $C_{\mathrm{m}} = 0.75\%$ and 1.00% PEO1M, where the triangular and circular data markers denote the storage (G') and loss (G'') moduli, respectively. Variations of **(c)** elastic modulus, G, and **(d)** zero-shear viscosity, η_0, with polymer concentration, C_{m}. The dashed lines in panel d denote the estimates of the entanglement concentration, C_{e}, for both PEO1M (red) and PEO4M (blue). In panels a, c, and d, the discrete markers denote the mean of at least three independent experimental realizations while the error bars indicate $\pm$ one standard deviation.

Rheological characterization

Rheological measurements were performed on a stress-controlled rotational rheometer (MCR 501, Anton Paar GmbH) using a cone-and-plate geometry (1° angle, 50 mm diameter, and mean gap of 0.1 mm). A solvent trap containing a 40% (by mass) IPA-water solution was employed to prevent evaporation during the measurements. All measurements were performed at 19 °C and in triplicate.

The shear viscosity (η) as a function of the shear rate ($\dot{\varepsilon}$) for different polymer concentrations (C_{m}) is presented in figure S2a. The focus of the present work is on elucidating the role of viscoelasticity on the Marangoni bursting behavior. However, dissolved polymers may also impart shear thinning behavior (as also observed at high C_{m} in figure S2a), which further complicates the dynamics of an already complex phenomenon. Hence, in the present study, we limit C_{m} such that the polymeric liquids behave predominantly as Boger fluids[3], i.e. their shear viscosity is independent of the shear rate (although there is departure from Boger fluid-like behavior at high C_{m} for high shear rates, as seen in figure S2a). The corresponding zero-shear viscosities (η_0) can also be extracted from the shear viscosity vs. shear rate curves for the different polymer concentrations.

Frequency (ω) sweep measurements were performed at an amplitude of 10% (determined after thorough amplitude sweep measurements) to determine the linear viscoelastic storage (G') and loss (G'') moduli of the polymeric liquids used in the present work. All measurements were performed at 19 °C. The measurements

for two representative concentrations ($C_{\rm m} = 0.75\%$ and 1.00% PEO1M) are shown in figure S2b. While no crossover between G' and G'' is observed within the frequency (ω) range shown in figure S2b, the experimental trends suggest the crossover frequency to be $\mathcal{O}(100\,\text{Hz})$—consistent with prior measurements[4,5] of relaxation times of PEO solutions of comparable concentrations. However, at such high frequencies, inertial artifacts in the frequency sweep measurements became unavoidable, resulting in unreliable data. Hence, extensional thinning measurements were relied upon to accurately determine the relaxation times and elastic moduli of the polymeric solutions.

The relaxation times of the polymer solutions (τ) were measured from the extensional thinning of liquid filaments in a pendent droplet configuration[6–9], which is known[4,5,10] to provide accurate estimates of the relaxation times for stretching filaments of PEO solutions. All measurements were performed at 19 °C and in triplicate. Knowing the zero-shear viscosity (η_0, which is also the shear viscosity at all shear rates for Boger fluids) and the relaxation time (τ) for different polymer concentrations allows for the estimation of the elastic modulus: $G = \eta_0/\tau$. The variation of the elastic modulus (G) with the polymer concentration ($C_{\rm m}$) is shown in figure S2c. The elastic modulus (G) increases with increasing polymer concentration ($C_{\rm m}$) for both PEO1M and PEO4M, with steeper increases observed for the higher molecular weight polymer (PEO4M).

The entanglement concentration ($C_{\rm e}$) is determined to be the polymer concentration at which the zero-shear viscosity (η_0) of the polymer solution rapidly increases[11]. The variation of zero-shear viscosity (η_0) with polymer concentration ($C_{\rm m}$) is shown in figure S2d. For the polymers used in the present study, the entanglement concentrations were determined to be $\approx$ 0.25% (by mass) and 0.18% (by mass) for PEO1M and PEO4M, respectively (dashed lines in figure S2d).

Spreading dynamics, droplet lifetime, and fragmentation time

The dynamics of Marangoni-stress driven spreading processes can be captured from simple scaling arguments[12–14]. In the present work, a water-IPA droplet (with or without dissolved polymers, since the presence of dissolved polymers were observed to not have any significant impact on the spreading dynamics; see figure 1a), with IPA mass fraction ϕ, spreads on an oil layer of density $\rho_{\rm o}$, with an instantaneous spreading radius of $R(t)$. The alcohol surface concentration, Γ, scales as[12,14] $\Gamma \sim \phi/R^2$, resulting in a Marangoni stress given by[14]

$$\sigma_{\rm Marangoni} \sim \frac{A\Gamma}{R} \sim \frac{A\phi}{R^3}\,, \tag{S1}$$

where $A = -{\rm d}\gamma/{\rm d}\Gamma$ represents the gradient of surface tension, γ, with surface concentration, and can be considered to be equivalent to a surface activity[14]. This spreading liquid front experiences a viscous resistance from the oil substrate, and the corresponding viscous stress is given by

$$\sigma_{\rm viscous} \sim \frac{\eta_{\rm o} R}{t\,h_{\rm o}}\,, \tag{S2}$$

where the viscous boundary layer is considered to penetrate the entire oil layer thickness, $h_{\rm o}$, at time scales much faster than the characteristic spreading time scale (see also the derivation for equation (11)). If the alcohol concentration, ϕ, is sufficiently large, as in the present case, A behaves as a constant in time. Hence, combining equations (S1) and (S2) results in

$$R \sim t^{1/4}\,, \tag{S3}$$

which has also been observed in similar experimental configurations[14,15]. The trends described by the dashed lines in figure S3a are also consistent with this $R \sim t^{1/4}$ scaling, but given the limited range of our data, we refrain from claiming any scaling relationship at this point.

The variation of the normalized spreading radius, $R/R_{\rm max}$, with normalized spreading time, $t/t_{\rm exp}$, is shown in figure S3b for different polymer concentrations. All experimental datapoints tend to collapse on one master curve, which was also observed in prior studies with Newtonian fluids[16].

The variation of the normalized fragmentation time, $t_{\rm frag}/t_{\rm exp}$, with polymer concentration, $C_{\rm m}$, is shown in figure S3c. The normalized fragmentation time increases with increasing $C_{\rm m}$, thus emphasizing the relative delay in self-emulsification, and thus the extended lifetime of the mother droplet, as the polymer concentration increases.

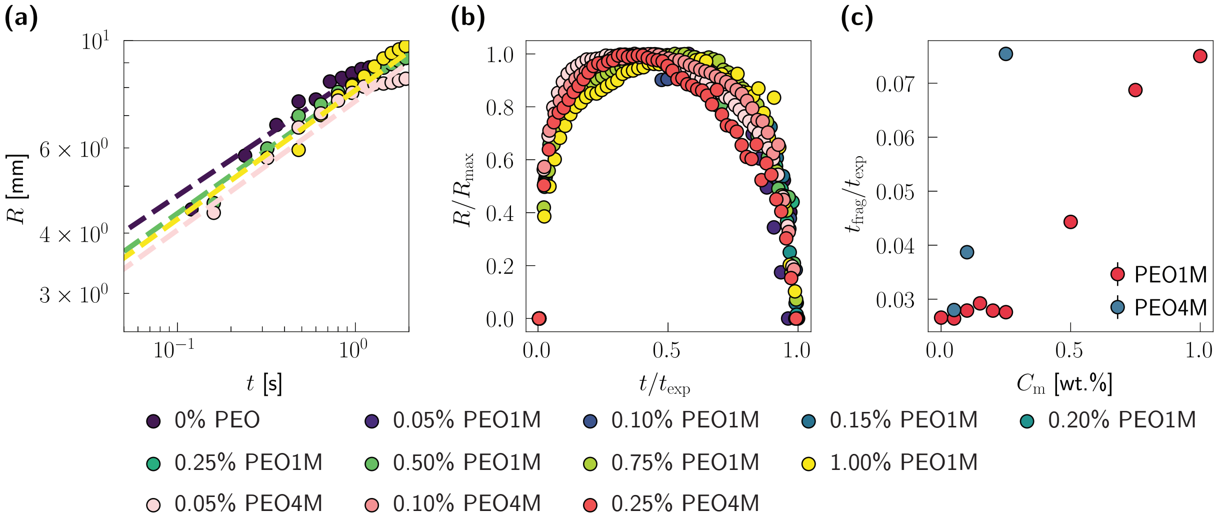

Figure S3: **Spreading dynamics, normalized spreading, and fragmentation times. (a)** Early time spreading dynamics of the mother droplet, where the dashed lines denote $R \sim t^{1/4}$. **(b)** Variation of normalized spreading radius, $R/R_{\rm max}$, with normalized spreading time, $t/t_{\rm exp}$, for different polymer concentrations, where all experimental datapoints tend to collapse on one master curve. **(c)** Variation of the normalized fragmentation time, $t_{\rm frag}/t_{\rm exp}$, with polymer concentration, $C_{\rm m}$, which exemplifies the relative delay in self-emulsification as the polymer concentration increases. The discrete markers in panel c denote the mean of at least three independent experimental realizations while the error bars indicate $\pm$ one standard deviation.

Number of fingers decreases with increasing polymer concentration

The temporal variation of the mean finger number, $n_{\rm finger}$, for different polymer concentrations is shown in figure S4. To filter out the misdetection of fingers, we only consider experimental snapshots containing at least 10 fingers. Moreover, for $t \lessapprox 3$ s, the finger dimensions fall below the reliable detection limit of the image processing algorithm, thus underestimating the number of fingers. Consequently, we observe a false convergence of $n_{\rm finger}$ towards 0 at time $t = 0$. However, our experiments indicate that the fingering instability develops almost instantaneously. Hence, figure S4 underestimates the number of fingers for $t \lessapprox 3$ s. A similar underestimation was also observed towards the end of each experiment, i.e. for $t \gtrapprox 8$ s. To mitigate this, we focus on the time-span where the finger detection is robust, denoted by the shaded area in figure S4. Within this time-span, the number of fingers gradually decreases with time for all polymer concentrations. However, since the spreading radius, R, of the mother droplet also decreases within this time-span (as seen in figure 2a), the instability wavelength, $\lambda = 2\pi R/n_{\rm finger}$, remains fairly constant (as seen in figure 3a). Additionally, the number of fingers is observed to decrease with increasing polymer concentration.

Viscoelastic control of self-emulsification is polymer-independent

The broader applicability of this method of leveraging polymer-induced viscoelasticity to control Marangoni-driven self-emulsification requires the method to be independent of the molecular structure of the dissolved polymer (i.e. polyethylene oxide (PEO) in the main manuscript). This was confirmed by performing Marangoni-bursting experiments with polyvinylpyrrolidone (average molecular weight $\approx 360 \times 10^3$ Da, Sigma-Aldrich, henceforth referred to as "PVP") as the dissolved polymer, instead of PEO. The IPA concentration in the resulting polymeric solution (of IPA, water, and PVP) was kept constant at 40% (by mass) while the PVP concentration ($C_{\rm PVP}$) was varied between 1.0% and 5.0%. The materials characterization methods, experimental protocols, and analysis techniques were identical to the ones described for PEO solutions in the main manuscript (see movie SM5 for typical experimental realizations of Marangoni-driven self-emulsification with PVP solutions).

The densities of the PVP solutions were measured to be 936 and 953 kg/m^3 for the 1.0% and 5.0% concentrations, respectively. Extensional thinning measurements indicated that while the 5.0% PVP solution had a relaxation time (τ) of 1.23 ms, no prominent elastocapillary thinning was observed for the 1.0% PVP solution. Hence, the 1.0% PVP solution was adjudged to have a Newtonian response. Shear rheology (see figure S5a) also indicated that both the 1.0% and 5.0% PVP solutions behaved as Boger-like fluids, although a slight shear-thinning was observed at high shear rates for the 5.0% PVP solution. Measurements (see figure S5b) further confirmed the drop-oil ($\gamma_{\rm da}$) and drop-air ($\gamma_{\rm da}$) interfacial tension coefficients to be effectively

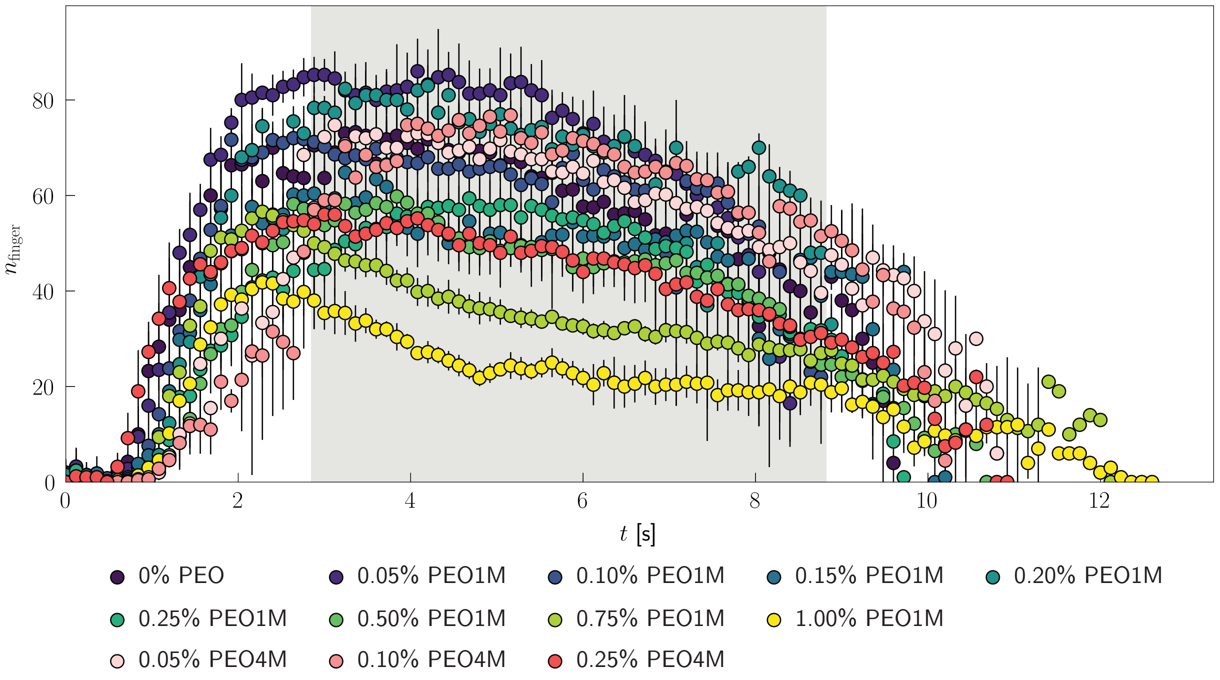

Figure S4: **Number of fingers.** Temporal variation of the number of fingers, n_{finger}, for different polymer concentrations. The shaded area denotes the time-span corresponding to stable finger detection. The discrete markers denote the mean of at least three independent experimental realizations while the error bars indicate $\pm$ one standard deviation.

independent of the polymer concentration, C_{PVP}, at $\gamma_{\text{da}} = 24.5$ mN/m and $\gamma_{\text{do}} = 4$ mN/m, respectively.

The temporal evolution of the spreading front radius, R, follows similar dynamics as PEO-containing droplets (see figures S5c and 2a)—first increasing till reaching a maximum and subsequently decreasing, the latter being accompanied by a spontaneous destabilization of the droplet periphery, resulting in self-emulsification (see movie SM5 for the corresponding movies). Here as well, the introduction of viscoelasticity—achieved via the increment of the concentration of dissolved PVP—delays the onset of self-emulsification, as shown in figures S5d, 2d, and S3c.

Similar to the PEO-containing droplets, fragmentation here also proceeds with the formation of protruding fingers from the periphery of the spreading droplet, where the number of fingers (n_{finger}) first increases with time, and then gradually decreases (see figures S5e and S4). However, the finger detection algorithm also suffers from the same shortcoming at early times ($t \lessapprox 3$ s) as the one described for PEO-containing droplets (figure S4), resulting in an underestimation of the number of fingers at early times. The number of fingers was also observed to decrease with increasing PVP concentration, mirroring the trend observed for PEO-containing droplets (see figures S5e and S4). A similar correspondence between the PEO- and PVP-containing droplets was also observed for the temporal variation of the instability wavelength, λ, which remains practically constant in time, but this constant value increases with increasing polymer concentration (see figures S5f and 3a).

The finger length, l_{f}, distribution exhibit a gradual broadening with time, as seen in figures S5g-i and S5g-ii. The broadening effect is also more pronounced at higher PVP concentrations. Moreover, both of these observations are consistent with our observations for PEO-containing droplets (see figures 4a-i – 4a-iii). The similarities with PEO-containing droplets in the temporal evolution of distributions also extend to the daughter droplet radius (r_{d}) distribution (see figures S5h-i and S5h-ii), where a shift towards larger droplet sizes at later times is also observed. The daughter droplet radius distributions are also well-fitted by the log-normal distribution here (dashed lines in figures S5h-i and S5h-ii), consistent with the PEO-containing droplets (see figures 5a-i – 5a-iii). The variations of the distributions of l_{f} (figure S5i) and r_{d} (figure S5k) with PVP concentration (C_{PVP}) also show the same trend as PEO-containing droplets (see figures 4b-i, 4b-ii, 5b-i, and 5b-ii). Hence, we can conclude that leveraging polymer-induced viscoelasticity to control the dynamics of Marangoni-driven self-emulsification at fluid-fluid interfaces is independent of the molecular structure of the dissolved polymer.

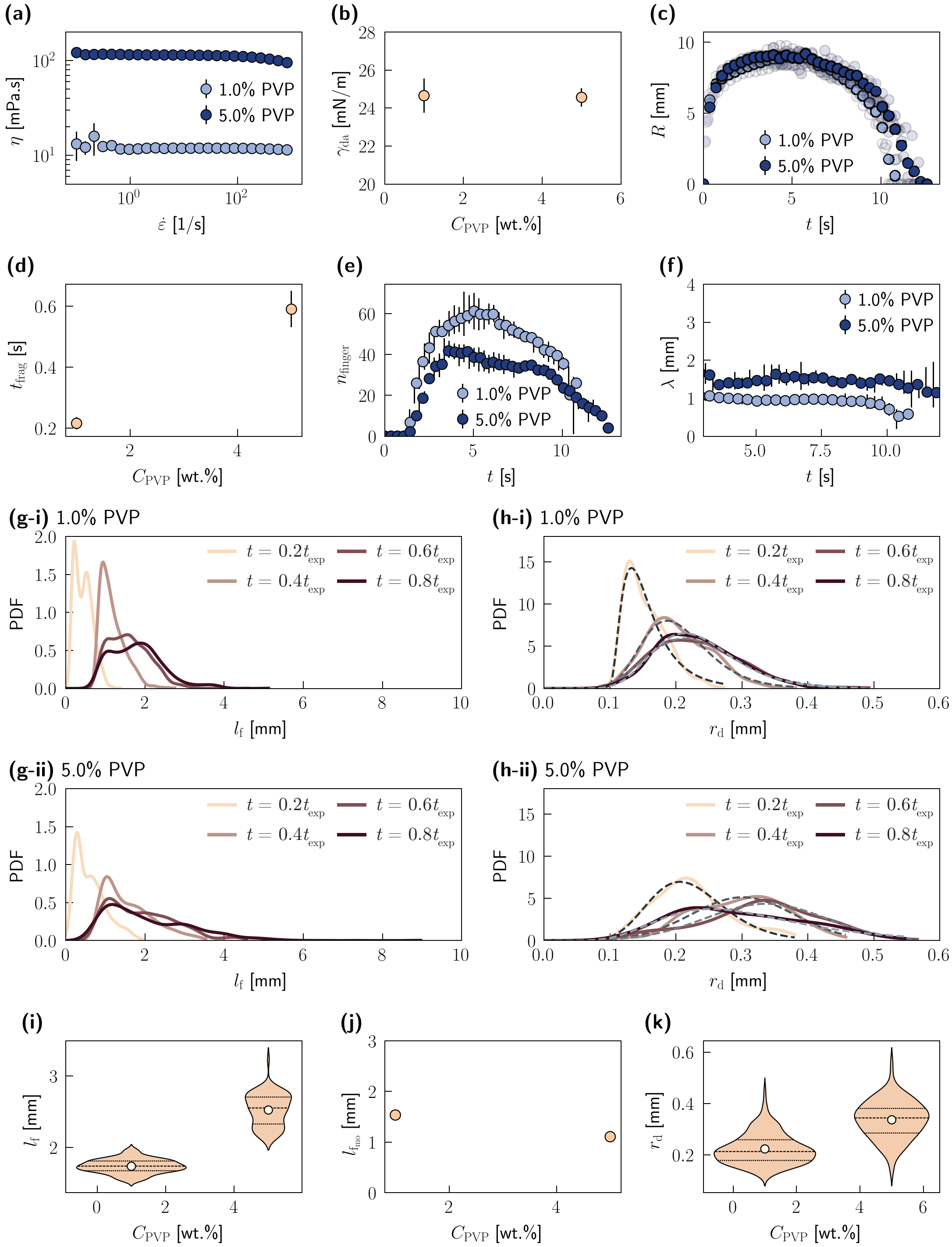

Figure S5: **Marangoni-driven self-emulsification of PVP-containing droplets.** **(a)** Variation of shear viscosity, η, with shear rate, $\dot{\varepsilon}$, for two different PVP concentrations. **(b)** Variation of the drop-air interfacial tension coefficient, $\gamma_{\rm da}$, with PVP concentration, $C_{\rm PVP}$. **(c)** Temporal evolution of the spreading front radius, R, for two different PVP concentration. The transulucent symbols denote at least three independent experimental realizations per polymer concentration while the opaque symbols indicate the mean for each polymer concentration. **(d)** Time of incipence of self-fragmentation, $t_{\rm frag}$, for two different PVP concentrations. Temporal variation of **(e)** the number of fingers, $n_{\rm finger}$, and **(f)** the instability wavelength, λ, for two different PVP concentrations. The discrete markers in panels a, b, and d – f denote the mean of at least three independent experimental realizations while the error bars indicate $\pm$ one standard deviation. Distributions of the **(g)** finger length, $l_{\rm f}$, and **(h)** daughter droplet radii at different time instants, and for two different PVP concentrations. The dashed lines in panels h-i and h-ii denote the corresponding log-normal distribution fits. **(i)** Distributions of finger length, $l_{\rm f}$, at $t = 0.5\,t_{\rm exp}$ for two different PVP concentrations. **(j)** Variation of the characteristic finger length, $l_{\rm f_{mo}}$, at $t = 0.6\,t_{\rm exp}$ for two different PVP concentrations. **(k)** Distributions of daughter droplet radius, $r_{\rm d}$, at $t = 0.5\,t_{\rm exp}$ for two different PVP concentrations. The discrete datapoints in panels i and k denote the mean value of each distribution. See movie SM5 for the corresponding movies.

Supplementary movies

Movie SM1: Marangoni-driven self-emulsification of a water-IPA droplet containing 0.10% PEO1M.

Movie SM2: Marangoni-driven self-emulsification of a water-IPA droplet containing 0.50% PEO1M.

Movie SM3: Spreading dynamics during Marangoni-driven self-emulsification of a water-IPA droplet ($C_{\mathrm{m}} = 0\%$).

Movie SM4: Marangoni-driven self-emulsification of a water-IPA droplet containing 0.90% PEO4M.

Movie SM5: Marangoni-driven self-emulsification of water-IPA droplets containing 1.0% and 5.0% PVP.

REFERENCES

[1] A. Daerr and A. Mogne. Measuring liquid surface tension through the pendent drop method: Description of a measurement bench and an imagej plugin. *J. Open Res. Softw.*, 4:1–12, 2016.

[2] J. Schindelin, I. Arganda-Carreras, E. Frise, V. Kaynig, T. Pieztsch, S. Preibisch, C. Rueden, S. Saalfeld, B. Schmid, J.-Y. Tinevez, D. J. White, V. Hartenstein, K. Eliceiri, P. Tomancak, and A. Cardona. Fiji: an open-source platform for biological-image analysis. *Nat. Meth.*, 9:676–682, 2012.

[3] D. F. James. Boger fluids. *Annu. Rev. Fluid Mech.*, 41:129–142, 2009.

[4] U. Sen, D. Lohse, and M. Jalaal. Elastocapillary Worthington jets. *arXiv:2207.07928*, 2022.

[5] M. Li, Y. Saade, S. Zaleski, U. Sen, P. Kant, and D. Lohse. Viscoelasticity reduces the droplet size in mucosalivary film fragmentation during intense respiratory events. *arXiv:2502.05105*, 2025.

[6] A. Deblais, K. P. Velikov, and D. Bonn. Pearling instabilities of a viscoelastic thread. *Phys. Rev. Lett.*, 120:194501, 2018.

[7] S. Sur and J. Rothstein. Drop breakup dynamics of dilute polymer solutions: Effect of molecular weight, concentration, and viscosity. *J. Rheol.*, 62:1245–1249, 2018.

[8] W. Mathues, S. Formenti, C. McIlroy, O. G. Harlen, and C. Clasen. CaBER vs. ROJER – Different time scales for the thinning of a weakly elastic jet. *J. Rheol.*, 62:1125–1153, 2018.

[9] A. Deblais, M. A. Herrada, J. Eggers, and D. Bonn. Self-similarity in the breakup of very dilute viscoelastic solutions. *J. Fluid Mech.*, 904:R2, 2020.

[10] U. Sen, C. Datt, T. Segers, H. Wijshoff, J. H. Snoeijer, M. Versluis, and D. Lohse. The retraction of jetted slender viscoelastic liquid filaments. *J. Fluid Mech.*, 929:A25, 2021.

[11] Y. Heo and R. G. Larson. The scaling of zero-shear viscosities of semidilute polymer solutions with concentration. *J. Rheol.*, 49:1117–1128, 2005.

[12] O. E. Jensen. The spreading of insoluble surfactant at the free surface of a deep fluid layer. *J. Fluid Mech.*, 293:349–378, 1995.

[13] A. B. Afsar-Siddiqui, P. F. Luckham, and O. K. Matar. Unstable spreading of aqueous anionic surfactant solutions on liquid films. part 1. sparingly soluble surfactant. *Langmuir*, 19:696–702, 2003.

[14] S. T. Chan and E. Fried. Marangoni spreading on liquid substrates in new media art. *PNAS Nexus*, 3:pgae059, 2024.

[15] M. A. Hack, M. N. van der Linden, H. Wijshoff, J. H. Snoeijer, and T. Segers. Ring-shaped colloidal patterns on saline water films. *J. Colloid Interface Sci.*, 673:788–796, 2024.

[16] L. Keiser, H. Bense, P. Colinet, J. Bico, and E. Reyssat. Marangoni bursting: evaporation-induced emulsification of binary mixtures on a liquid layer. *Phys. Rev. Lett.*, 118:074504, 2017.
